# Hospital Administration and Organisation in South Africa

**Published:** 1911-01-07

**Authors:** 


### SPECIAL INSTITUTIONAL ARTICLES.

### HOSPITAL ADMINISTRATION AND ORGANISATION IN SOUTH AFRICA.

### THE QUESTION OF HOSPITAL ABUSE AND ITS PREVENTION.

At the recent Medical Congress held at Cape Town during the first week in November last, some important resolutions with reference to the hospital question in the new Dominion were submitted and passed, and an interesting paper on the general subject of "Hospital Administration and Organisation" was read by Sir Kendal Franks. Through the courtesy of Dr. Darley-Hartley, M.R.C.P., Editor of the *South African Medical Record*, we have been permitted to take over the following summary of the paper, and the discussion that followed its reading, from a recent issue of the *Record*.

Sir Kendal Franks commenced by remarking that it was high time the subject of hospital administration and organisation was properly tackled by the medical profession, for it had been neglected too long, largely through the fault of the medical men, who in South Africa seemed to have tacitly allowed themselves to be superseded by laymen, so far as hospital administration was concerned. In South Africa laymen knew comparatively little about hospital administration. In a country like England, where there was a large number of well-trained lay experts in hospital management, no harm was done; but in South Africa, where the only men who knew anything about the subject were medical men, they had naturally got on to entirely wrong lines.

#### The Voluntary System in South Africa.

Remarking that the want of cohesion in the profession was the main cause of its not having properly asserted itself in this connection, he mentioned incidentally that this lack of cohesion had the further evil result of creating among the laity a marked suspicion of medical interference. It was painful to see the lack of respect towards medicine as compared with the great respect shown towards a profession like the law. He expressed his opinion that in South Africa it was impossible to depend wholly, or even mainly, on voluntary contributions, or, at least, they certainly could not do so in the Transvaal. He passed on to give a history of the Johannesburg Hospital, and explained that this hospital was now entirely a Government institution, the Government having appropriated the proceeds of a capitation tax paid by employers of labour on behalf of their native employees—a tax which brought in very much more than was expended on the hospital.

He advocated this system of supporting hospitals by similar contributions paid by employers of labour. This was not the same as the nationalisation of hospitals, which he would regard as a great calamity. Patients should be primarily admitted because they were poor. He did not advocate the Socialistic view that everybody was entitled to hospital treatment, and that the profession should resist the introduction of the thin end of that wedge, but at the same time he advocated the exaction of payment from those who could contribute something towards the expense of that treatment.

#### Schools of Pauperism.

The English system, by which no payment was expected, had converted the hospitals into schools of pauperism aided by medical sentimentality. He described the American system as a great improvement upon that of England. There free beds existed, but their number was strictly limited and very much smaller in proportion to the total number of beds than in England or in the Continental countries. Moreover, a very strict system of supervision fixed upon the applicant for the free bed the onus of proving that he was quite unable to pay for his necessities. This was the exact converse of the English system. This question of hospital abuse had for some years excited the earnest attention of the profession and hospital administrators in England, and almoners had been appointed at some of the large hospitals; but this method, although it had been extensively tested, had not effected much good, and had perpetuated the old error of regarding the hospitals as primarily free, and of laying on the managers the onus of proving that the person had means. A very interesting discussion had taken place at the Australasian Congress of 1908, in a country where the conditions very closely resembled those in South Africa. The result was three resolutions—one providing that no payment should be taken at public hospitals; the second that a person desiring admission should fill up and sign a declaration of his inability to pay; and the third that such declaration should in all cases be supported by certificates from the applicant's medical attendant. The opinion there was in favour of the establishment of three classes of hospitals—respectively public general hospitals for free patients, private self-supporting nursing homes for the well-to-do, and intermediate hospitals, partly supported by the benevolent, for that class who, whilst not indigent, were not able to pay the full expenses of their treatment. It was contemplated that both of the latter classes should pay medical fees. He went on to speak of the present system in South Africa, which was sweating the profession. In protesting against it medical men were accused of self-interest, but, as a matter of fact, their interest was the public interest. If of our charity we gave the most valuable part to the care of the sick, we certainly ought to have some voice in the mode in which that charity is to be dispensed. Detailing the system in vogue at the Johannesburg Hospital, he described a scheme, for which General Smuts was at least technically responsible, under which it was proposed to reduce the rates charged for the paying beds to the figure of ten shillings, and at the same time to insist that the patients paying this sum should be entitled to the free attendance of the honorary hospital staff. The gist of this idea was to make the paying beds as cheap as possible, and thereby reduce the number of applications for free beds, and to do all this at the expense of the unpaid medical staff. The great evil of the Johannesburg Hospital was the mixture of free and paying patients in the same wards, and he advocated that certain pavilions of that hospital should be set entirely aside for free patients, and others for the contributing ones. The well-to-do patients, able to pay at remunerative rates, should be provided for in private nursing homes. In Johannesburg private homes found it extremely difficult to compete with the hospital; but with that competition withdrawn numbers of them, of an excellent type, would arise in Johannesburg or in any large town. He regarded subscribers' letters of recommendation as worse than useless. The almoner system was only a partial success. Letters from medical attendants were likely to be very good. There was a common error to the effect that members of hospital staffs, even though unpaid, obtained a very valuable *quid pro quo* in the shape of professional prestige, and it was often asserted, quite unblushingly by certain members of Hospital Boards, that some members of the staff surreptitiously took fees from free patients—a most scandalous assertion. There was no doubt that a position on a hospital staff was in England a most valuable assistance to a consultant who had a reputation to make, but that certainly was not the case in South Africa.

Dealing with the fact that the profession was mostly not represented in South Africa on the Hospital Boards, he stated that this was most unfair, and should be remedied, and he made some scathing remarks with reference to recent action taken by the Board of the Frere Hospital at East London.

### DISCUSSION.

Dr. Darley-Hartley stated that he should not have spoken upon the very valuable paper by Sir Kendal Franks but for the fact that he had some time ago been Chairman of the Special Committee appointed by the Western Branch of the British Medical Association for the purpose of going into this subject. That Committee had gathered together a large mass of material, and had prepared a valuable report. Its conclusions in the main were identical with those arrived at by the Australasian Medical Congress, the most important being the entire separation of hospitals for free patients from those of paying or partly paying ones. Personally he regarded the reservation of State-aided hospitals for free patients, and no others, to be the most important thing. The moment paying or partly paying patients were mixed with free patients in the same hospital one had the germ of the Socialistic idea: that every inhabitant of the country, whatever might be his rank in life, was entitled to hospital attendance so long as he paid according to his means. The end of this would be that in all large centres every serious illness—certainly every serious surgical illness—would be treated in hospital. The only men who would get any practice in the higher classes of work would be salaried medical servants of a commercialised hospital, probably salaried servants worked to such an extent that they had little time for thought or research. The physician and surgeon would disappear, and the general practitioner would become a man of about the same stamp as the British apothecary of one hundred and fifty years ago—simply the treater of minor ailments. The most difficult problem to deal with certainly was provision for the large class who, whilst not eligible for free treatment, were not able to pay the full expense of at least long illnesses or major surgical operations. But this problem was not as difficult as it appeared at first sight. Once shut the doors of the general hospital to this class of patient, the benevolent public would come forward and subsidise, or at least guarantee, these intermediate hospitals, which he believed could be made, if modestly and inexpensively worked on a system of large wards, in most cases self-supporting, at rates quite within the means of this class of patient. He described the Swiss system of such hospitals, which were sometimes able to pay their way on four francs per day. Medical fees in such hospitals should be paid to any attendant whom the patient selected. Of course, they must be at a reduced rate, and in some cases the medical attendant would remit them altogether, which was nothing more than they all did in private practice, but they ought to be allowed to be their own judges as to whether they would remit them or not.

### Pauper Patients.

Dr. Casalis, generally agreeing with the contentions of Sir Kendal Franks, considered that hospital appointments were of practically no value to practitioners.

Dr. Rogers combated Dr. Casalis' views on this point, thinking that the experience gained was of considerable value. He emphasised strongly the advantage of having members of the staffs elected by a committee composed partly of the staff itself and partly of representatives of the outside profession.

Dr. Hewat stated that they were unanimous in their willingness to give gratuitous attendance to really poor people, and then moved a resolution to the effect that State-aided hospitals for pauper patients should be provided, and entirely separate from hospitals receiving payment.

Dr. John Brown suggested the elimination of the word "pauper," which had an objectionable sound.

Dr. Hewat expressed himself as quite willing to substitute a better word.

The President (Dr. A. B. Ward) was entirely in accord with the resolution, but thought that hospitals for the poor should be supported by the State. There was great difficulty in obtaining subscriptions from the public. He thought, however, that Dr. Hewat's resolution must refer only to large centres, and that there such hospitals should be entirely free. Paying hospitals should not even be under the same roof. The mixture of paying and non-paying patients in the same wards, or even in the same building, was a great grievance, and caused much jealousy between the two classes.

Dr. Ireland, speaking from the standpoint of his own town, Cradock, said that it was impossible to apply Dr. Hewat's principle to small places, inasmuch as there was no room for separate institutions, and all classes of patients must be treated in the general hospital.

Dr. Hewat stated that he entirely recognised Dr. Ireland's contention. In cottage hospitals in small places all classes must be received. He was quite willing to modify his resolution by the insertion of the words "in centres sufficiently large."

Dr. Balfe asked if it was intended that the resolution should apply to existing hospitals.

Dr. Hewat replied that it was intended to be their expression of opinion, and circumstances would determine as to how far the authorities would apply it to existing hospitals.

Dr. Darley-Hartley seconded Dr. Hewat's resolution.

Dr. Ireland stated that at Cradock all patients went under one roof. Free patients and those paying under five shillings paid no medical fees; those over five shillings were liable to such fees; whilst the well-to-do were given private wards at 10s. 6d., and, of course, paid their own medical attendant. Owing, perhaps, to their having a very energetic secretary, there was very little hospital abuse and the hospital paid its way.

### Support for the Voluntary System.

Dr. Elliott said that he would be reluctant to affirm definitely that general hospitals should be entirely supported by the State, which was the German system. The British system was that the hospitals were entirely dependent upon the benevolent public, and he thought that this was one of the glories of England.

Dr. Ireland raised the question of railway patients, for whom at present 3s. 6d. per day was paid by the Sick Fund.

Dr. Hewat pointed out that it was impossible to legislate for this. If the Government, in return for the contribution to hospitals, insisted on their servants being admitted at 3s. 6d. per day, it was impossible at present to resist that contention. Sir Kendal Franks said that their difficulty at Johannesburg was that they would shortly have five hundred beds in the hospital, and it would be impossible to fill these with free patients if they took all paying patients to a separate institution, although he believed that the principle of separation was a sound one for future application. His plan was to exclude full-paying patients from the hospital entirely, and to treat partially paying ones in separate pavilions of the same institution.

Dr. Reynolds asked whether Government patients at 3s. 6d. a day were to be considered free or partially paying patients. Were they to be treated by the honorary staff without fees, or not?

Dr. Darley-Hartley said that difficulty was easily got over by the paid Government medical officers bringing Government patients into the hospital and attending them there. That was what was done in East London.

Dr. Elliott thought that railway people should be taken into an intermediate hospital.

Dr. Fuller did not think Sir Kendal Frank's principle compatible with the resolution, and he said the expenses of administration would be enormously increased if you had separate wards of the same institution. He would prefer the general hospitals being quite free.

### The Natal System.

Dr. Balfe detailed the system in vogue in Natal. They had tried hard to separate free patients from paying or part-paying ones, but had not been able to manage it. What happened in their small cottage hospitals was that the district surgeon attended free patients in the hospital gratuitously, but then he received a Government salary. Fully paying patients were liable for fees. The principle exemplified in the resolution was a sound one, and it was for the Government, or the managers of the institution, to settle how it was to be carried into effect.

Dr. Darley-Hartley suggested that the difficulty raised by Dr. Ireland could easily be met by the insertion of the words "except in cases of accident or emergency." They would thus lay down the principle that all hospitals would be free, subject to this limitation, and that limitation would meet the case of the small places like Cradock, because if there was no home or intermediate hospital in the place the case would become one of emergency.

Dr. Hewat's proposition was carried, with some verbal amendments.

Dr. Hewat moved a resolution to the effect that where satisfactory nursing homes exist which are available for any registered practitioner, patients able to pay for treatment at such homes shall not be admitted to State-aided hospitals. This was carried.

### Paying Patients.

Dr. Hewat then moved a resolution providing that, to meet the cases of patients unable to pay full fees, either intermediate hospitals or separate departments of existing hospitals should be set aside; medical men be entitled to charge fees for attendance whenever patients contributed more than the daily cost of maintenance; these hospitals to be partly supported by Government and partly by private subscriptions.

Dr. Darley-Hartley objected to Government dealing in any way with paying patients.

Sir Kendal Franks said that General Smuts' fixed idea was that the Government should provide hospitals for all classes of patients.

Dr. Keegan said that that was exactly what they wanted to avoid.

Sir Kendal Franks said that contributions from the benevolent public were unfair, as they fell entirely on a few.

Dr. Keegan said the subject had been fully gone into at Graaff-Reinet, where it had been found that the larger proportion of patients were the people who would go into an intermediate hospital, if such existed. A tax had been suggested, but the public resented it. He believed in keeping the Government as much as possible out of hospital administration, except as regards absolutely free patients. The local community should deal with the intermediate class.

Dr. Caporn supported Government dealing with both classes. The intermediate class were really the most deserving people.

Dr. Hewat withdrew the reference to support by the Government, and, after Sir Kendal Franks had seconded his proposition, it was carried.

### Pay and Private Wards.

Dr. Ireland proposed a resolution to the effect that where no nursing homes existed paying patients be admitted at fees from 3s. 6d. upward, and be considered as private patients, selecting their own medical attendant and paying his fees. To this an amendment was proposed that in small communities cottage hospitals be established providing for three classes of patients, if possible in separate departments, and that patients not paying more than 3s. 6d. per day be charged no medical fees.

Sir Kendal Franks thought that the minimum fee should be fixed by each hospital, and not laid down at 3s. 6d.

Dr. Darley-Hartley preferred Dr. Ireland's proposition, because it did not seem to contemplate any payment under 3s. 6d. being received. He thought that if a patient did not pay 3s. 6d. at least, he should be taken in for nothing at all. The man who paid a petty contribution of one shilling, or even sixpence, always went away with the comforting idea that he was beholden to nobody and had received no charity.

Sir Kendal Franks said that if a man could pay anything he should pay it, however small; otherwise you made a pauper of him.

On a division Dr. Ireland's proposition was carried.

Dr. Darley-Hartley moved a resolution to the effect that in allocating the three classes of patients the most satisfactory evidence was a certificate from a medical practitioner. He stated that a patient might be a fit subject for free attendance in a long illness when he was not entitled to it in a short one, and of the gravity of illnesses a medical man was the only competent judge; whilst as regards financial ability, knowing the people well, he was at least as good a judge as anybody else.

Sir Kendal Franks seconded this.

Dr. Hewat supported, stating that an additional argument was that, as medical men were the main bestowers of hospital charity, they should be allowed to say who were the fitting recipients.

The proposition was carried.

The wording of the resolutions as finally adopted was as follows :

1. "Whereas the medical profession is now, as ever, prepared to give free assistance to those unable to pay for same, this Congress affirms the advisability of the establishment of hospitals entirely or partly supported by Government for such cases only and in centres large enough to warrant such establishment. These establishments should be entirely apart from any hospital or department of same receiving payment in part or whole from its patients."

2. "Where satisfactory nursing homes exist which are available for any registered practitioner, patients able to pay for treatment at such homes shall not be admitted to State-aided hospitals."

3. "To meet the necessities of patients in large centres only able to pay part-fees for hospital accommodation, either intermediate hospitals be established or a separate department of a hospital be set aside for such purposes."

4. "That where no nursing home be available, patients be admitted to State-aided hospitals at fees from 3s. 6d. upwards, who shall be considered private patients, able to select their own medical practitioner, and who shall be responsible for his fees."

5. "That, in the classification of patients according to the foregoing resolutions, the most satisfactory form of evidence is a certificate from a medical practitioner."

